# The potential for patient-reported data and narratives to improve quality during emergency department boarding

**DOI:** 10.1093/haschl/qxaf138

**Published:** 2025-07-07

**Authors:** Kelly T Gleason, Kathryn M McDonald, Susan M Peterson, Diane Kuhn, Mark Schlesinger

**Affiliations:** Johns Hopkins University School of Nursing, Baltimore, MD 21205, United States; Johns Hopkins University School of Nursing, Baltimore, MD 21205, United States; Department of General Internal Medicine, Johns Hopkins University School of Medicine, Baltimore, MD 21205, United States; Department of Emergency Medicine, Johns Hopkins University School of Medicine, Baltimore, MD 21205, United States; Department of Emergency Medicine, Indiana University School of Medicine, Indianapolis, IN 46202, United States; William M. Tierney Center for Health Services Research, Regenstrief Institute, Indianapolis, IN 46202, United States; Department of Health Policy and Management, Yale University School of Public Health, New Haven, CT 06520, United States

**Keywords:** emergency department, patient-reported outcomes, diagnostic quality, quality improvement, patient experience surveys

## Abstract

Emergency department (ED) boarding, the holding of admitted patients in the ED due to unavailable inpatient beds, is a growing challenge linked to poorer patient outcomes. Traditional patient experience surveys, such as the ED Consumer Assessment of Healthcare Providers and Systems (ED CAHPS), exclude insights from boarded patients, whose experiences could inform quality improvement efforts. We collected data using the patient-reported outcomes to improve diagnostic experience in the ED instrument from all patients, whether discharged or admitted. Early findings show that longer ED stays correlate with lower patient-reported diagnostic quality scores, with the highest quartile of stay durations averaging 3.79 out of 5 compared to 4.10 in the lowest quartile. A separate nationwide survey of diagnostic experiences, which did not specifically ask about EDs or wait times, also highlighted the impact of long ED stays. Patient narratives emphasized the need for better communication during boarding, such as explaining the reasons for admission. These findings suggest that including boarded patients in ED CAHPS' sampling criteria and incorporating open-ended questions could provide valuable insights for improving ED boarding practices. This commentary emphasizes the value of patient-driven data in identifying actionable solutions to mitigate the challenges of ED boarding.

## Teaser

Surprisingly, patient experience data are not routinely collected from patients who must “board” in the ED after hospital admission when waiting for transfer to their hospital room. From recent research, lower patient-reported diagnostic quality scores correlate with longer ED stays, and patients report communication issues such as failure to explain the rationale for hospital admission during boarding. Continued research on and implementation of patient-driven metrics would provide critical insights to guide practice and policy related to ED boarding challenges.

## Background

Emergency department (ED) boarding, the practice of keeping a patient in the ED after the decision to admit them has been made due to the unavailability of a bed in the appropriate inpatient unit, has become more common in recent years.^1^ National data on 46.2 million hospitalizations from 2017 to 2024 showed that at its peak in January 2022, over 40% of patients were boarded more than 4 h.^2^ While the association between ED boarding and poor patient outcomes is well-documented,^3,4^ few efforts have been made to collect patient-reported data regarding ED boarding. Patients' capabilities to report information that can be used to improve quality of care are increasingly recognized^5^ and their insights may be important in guiding practice and policy solutions, both short-term and long-term, to ED boarding.

Despite the clear value of patient insights to improve ED boarding practices, patients who experienced boarding are largely excluded from efforts to collect patient-reported data after an ED visit. The most commonly used surveys of patient ED experiences, the ED Consumer Assessment of Healthcare Providers and Systems (ED CAHPS) surveys, are solely administered to patients who are discharged to home directly from the ED.^6^ The surveys that boarded patients do receive are thus about their hospitalization, with no focus on their ED experience. The general question used in ED CAHPS and other ED surveys to assess timeliness also would not capture boarding, regardless, as surveys typically capture how long it was before someone talked to them about the reason they were there, and how long it was before patients first received care.^6^ Patients might stay for over 24 h in the ED after first receiving care, and that long stay significantly impacts their experience and quality of care. In short, there is not currently a comprehensive way to understanding the impact of ED boarding on patients' perception of care.

## Emerging evidence of the impact of patient insights on ED boarding

Although we have limited understanding of how ED boarding—and the resulting prolonged wait times for all patients^7^—affects patient experience, existing evidence paints a concerning picture. EDs that have more extended boarding times have worse patient survey scores among their other patients who are directly discharged home, with these patients being less satisfied overall and less likely to recommend the hospital to others.^8^ One common aspect of ED boarding is ED hallway placement. Surveys of adult ED patients suggest that most prefer boarding in inpatient hallways over ED hallways, citing reasons such as lack of privacy and excessive noise.^9,10^ ED hallway placement is linked not only to lower likelihood of hospital recommendation and poorer overall ratings of care, but also to increased odds of patient elopement and return visits to the ED within 10 days.^11^

The patient-reported outcomes to improve diagnostic experience in the emergency department (PRIME-ED) survey is administered to patients regardless of whether they were discharged home or admitted to the hospital.^12^ Thus, while the survey does not focus on boarding specifically, the data collected include insights from patients who were boarded, and those data highlight what could be learned from patients to improve quality of care during boarding. PRIME-ED is being tested at 4 health systems in geographically distinct parts of the country. This pilot-testing generates qualitative and quantitative data relevant to boarded patients.

From these data, we see that length of ED stay affects patient-reported diagnostic quality of care, with the lowest length of stay quartile having an average score of 4.10 (on a 1–5 scale, with 5 being best) compared to an average score of 3.79 for the highest duration quartile. While a 0.31-point difference may seem modest, even a 0.2 change on a 5-point Likert scale is often considered clinically important in widely used patient experience surveys. Patient experience scores typically show a tight clustering at the high end, with most hospitals averaging between 4.0 and 4.5 on 1–5 scales, so a drop from 4.10 to 3.79 would move a hospital from above average to below average.^13^ Free-text comments from boarded ED patients have also identified opportunities for improved communication. For example, 1 patient comment explained: “*No one communicated with me. I was there 18 hours with chest pain and elevated blood pressure. No risks or diagnosis was given and I was passed off to the hospitalist*.”

Additional insights regarding learning from patient experiences boarding in the ED can be generated from the narratives elicited in a nationwide survey of patient experiences with diagnosis, agnostic to the site of the diagnosis.^14^ This survey captured patients' narrative accounts describing the impact of long ED stays on their quality of care, despite not explicitly asking about the ED or wait times. For example, 1 care partner recounted that her elder father developed delirium while boarding in the ED, which led to his other symptoms being masked and delaying his diagnosis after inpatient admission. ED boarding is a risk factor for developing delirium.^4^ She stated: “*It was very difficult for my father to communicate as he was delirious; all my sister and the doctors knew was that he was in pain.*” This data source also reveals many patient reports of problems and mistakes occurring because of long wait times in the ED. While not all long wait times are induced by boarding, boarding inevitably compounds wait times by tying up resources and space.

## Next steps

### Research

Larger studies specifically examining patient experiences during ED boarding are essential to better understand how boarding influences patients' perceptions of care. There are also opportunities to integrate existing data sources for new insights. For example, the Centers for Medicare and Medicaid Services collects data including time from the decision to admit a patient to that patient's placement in an inpatient bed.^15^

### Practice

Equipping patients with data on boarding and wait times to inform their decision on where to seek care may be an important step. For example, the Maryland Institute for Emergency Medical Services Systems has a public-facing website, which shares which EDs are on alerts. These alerts indicate when hospitals are temporarily overwhelmed and cannot safely manage lower urgency patients, and whether they have no electrocardiogram monitored beds available. While in many cases a patient experiencing an emergency health issue may not have a choice, this information can help a patient make an informed decision when hospitals are otherwise equally accessible and resourced. Given evidence that patients prefer boarding in inpatient rather than ED hallways, largely due to concerns about noise and privacy, hospitals should consider implementing full-capacity protocols, which use an all-hands-on-deck approach to relocate boarded patients out of the ED.^16^

### Policy

Having no data available on the experiences of boarded ED patients underscores what is lost by only administering ED CAHPS to patients who were not admitted to the hospital. Policy that encourages health systems to survey patients who are admitted to the hospital on their ED experience, instead of the current norm of only surveying patients discharged from the ED on their ED experience, is an important first step. More broadly, surveys that collect patients narrative accounts of their ED experiences, even if not focused explicitly on boarding, will identify more experiences that involve direct and indirect boarding effects and provide insights into how these negatively alter patient experiences and how those harms might be mitigated.

## Conclusion

Preliminary data unsurprisingly suggests that ED boarding negatively affects patient-reported outcomes and experiences. By incorporating insights from boarded patients, healthcare systems can better understand the causes and more effectively address the unique challenges posed by prolonged ED stays. These insights, combined with more comprehensive use of timeliness data and analysis with existing patient-reported outcome instruments, could guide targeted interventions to improve quality, communication, and overall care experiences for boarded patients. Patient-reported insights make feasible incentivizing health systems to improve ED boarding and reduce related care breakdowns.

## Supplementary Material

qxaf138_Supplementary_Data
